# Aluminum hybrid based on lignin from rice straw as a multifunctional additive for natural rubber composites with enhanced curing and thermo-oxidative stability

**DOI:** 10.1038/s41598-026-53139-6

**Published:** 2026-05-30

**Authors:** Salwa H. El-Sabbagh, Doaa S. Mahmoud, Khlood S. Abdel Zaher, Galal A. M. Nawwar

**Affiliations:** 1 Polymers and Pigments Department, National Research Centre, 33 El-Bohouth Street, Dokki, 12622 Giza, Egypt; 2 Green Chemistry Department, National Research Centre, 33 El-Bohouth Street, Dokki, 12622 Giza, Egypt

**Keywords:** Hybrid materials, Antioxidant, Composites, Rice straw, Natural rubber, Chemistry, Engineering, Environmental sciences, Materials science

## Abstract

The rising growth of agricultural waste poses significant challenges to environmental sustainability, community health, and commercial stability. However, the development of multifunctional bio-based additives that simultaneously provide reinforcement and antioxidant performance in natural rubber (NR) composites remains limited. This study examines the potential of an aluminum (lignin/silica/fatty acid) hybrid (Al(LSF)), derived from rice straw black liquor, as a dual-functional additive with reinforcing and antioxidant properties in natural rubber (NR) composites. The Al(LSF) hybrid was analyzed by XRF, SEM-EDX, TEM, particle size analysis, and zeta potential measurements. Morphology, curing behavior, mechanical properties, and thermo-oxidative aging resistance of Al(LSF) in NR matrices were also investigated. Results showed that Al(LSF) exhibited nanoscale characteristics enabling uniform dispersion in the NR matrix. The incorporation of Al(LSF) (1–4 phr) improved curing properties, as evident by the decreased optimum curing time by approximately 18.3% as compared to TMQ/NR composites. Tensile strength of NR composites increased by approximately 45.4%, and elongation at break reduced by approximately 26.4% at 1 phr loading of Al(LSF) hybrid. The improvement in thermo-oxidative aging resistance of NR composites with Al(LSF) could be observed from th increased aging coefficient from 0.43 to 0.55 (approximately 27.9%). NR composites reinforced with Al(LSF) displayed better mechanical strength and aging properties than those filled with conventional fillers such as silica and sodium bentonite, depending on hybrid loading. Al(LSF)/NR composites can be explored for applications requiring improved long-term thermo-oxidative aging resistance and mechanical strength, such as tires, conveyor belts, gaskets, shoe soles, and other rubber products used outdoors exposed to extreme environmental conditions. Therefore, Al(LSF) hybrid could be considered as a potential multifunctional bio-additive to enhance mechanical strength and thermo-oxidative stability of NR while valorizing agricultural waste.

## Introduction

The preparation of sustainable biomass-based functional materials from biomass has attracted great attention in various fields. In particular, the valorization of agricultural residues such as rice straw has emerged as an effective approach to address environmental and economic challenges. After pulping rice straw black, liquor is produced, a by-product rich in lignin, silica, phenolic compounds, fatty acids, and hemicellulose. Lignin, a major component, is a complex, cross-linked, and dark-colored biopolymer formed through the copolymerization of three phenylpropanoid units: syringyl (S), guaiacyl (G), and p-hydroxyphenyl (H). These units are interconnected via ether and carbon–carbon bonds, resulting in a highly irregular structure. Lignin also contains a diverse range of functional groups, including methoxy, hydroxyl, carboxyl, and carbonyl groups^1–3^. The phenolic hydroxyl groups in lignin react with the radical species responsible for oxidation, allowing lignin to act as an antioxidant^4–6^.

Rubbers are recognized as strategically important raw materials due to their unique entropy, elasticity and wide range of applications, including tires, seals, and gloves. However, diene-based rubbers containing unsaturated backbone structures are highly susceptible to thermo-oxidative aging, resulting in surface deterioration, increased hardness, and significant loss of mechanical performance^7,8^. The aging of rubber not only reduces service life but also leads to material waste and potential safety concerns. To overcome this limitation, antioxidants are typically incorporated during rubber compounding to delay degradation and extend service life^9^. Antioxidants are incorporated into natural rubber (NR) and synthetic rubber (SR) during mastication^10,11^. However, conventional antioxidants suffer from several limitations, including toxicity, volatility, and migration, which raise environmental and health concerns. Consequently, the development of non-toxic and sustainable anti-aging agents has become increasingly important. Lignin has been widely investigated as a bio-based antioxidant in rubber systems due to its phenolic structure and non-discoloring nature^12^. In addition, lignin incorporation into polymer matrices has been reported to enhance crosslinking, stabilization, and mechanical performance^13–16^. Lignin-based elastomers, including block copolymers, polyurethanes, and polyolefins, were prepared by Jiaxing et al. According to this study, changing the lignin ratio can improve Tg, tensile strength, elongation at break, and other characteristics of the matrix^17^. Natural rubber (NR) is one of the important elastomers that are utilized in the manufacture of many rubber products, including vehicle tires, belts, gloves, and medical equipment, accessories, and floor coverings^18^. Recent studies have demonstrated the potential of lignin-based and lignin–silica hybrid systems as sustainable multifunctional additives in rubber composites. For instance, silica–lignin hybrid fillers have been successfully incorporated into natural rubber matrices, showing improved surface properties, stability, and enhanced interaction between filler and polymer matrix^19,20^. In addition, lignin–silica nano-hybrids have been reported to significantly improve the mechanical and viscoelastic properties of natural rubber composites due to better dispersion and interfacial compatibility^21^. Moreover, hybrid systems based on bio-silica and lignin nanoparticles have demonstrated simultaneous reinforcement and antioxidant performance, contributing to enhanced tensile strength, abrasion resistance, and aging stability^22^. These findings highlight the growing interest in developing multifunctional bio-based hybrid materials for advanced rubber applications; however, their performance in complex multi-component hybrid systems remains insufficiently explored.

Previous studies have investigated the utilization of hybrids derived from rice straw black liquor as effective functional additives in rubber composites. These hybrids combine organic components (lignin and fatty acid) with inorganic/metallic components (silica and metal ions), resulting in a multifunctional material.

Abdel Zaher et al. studied the utilization of Ca(lignin/silica) prepared from rice straw in styrene-butadiene (SBR) composites as antioxidants the resulting SBR was found to have improved physical properties compared with SBR containing a commercial antioxidant (TMQ or IPPD)^2^. In addition, Abdel Zaher et al. studied the preparation of a zinc (lignin/silica/fatty acid) (Zn LSF) hybrid derived from rice straw and its utilization as a green antioxidant and activator in natural rubber composites, the results indicate that the physico-mechanical properties of natural rubber loaded with 7 phr of the Zn LSF complex improved compared with natural rubber containing the commercial activator ZnO/stearic acid^3^. Also, Mahmoud et al. studied the blending of natural rubber with an aluminum (lignin/silica/fatty acid) hybrid, which led to flexible, conductive rubber with higher electrical conductivity^23^.

Nevertheless, broader comparisons with recent international studies on bio-based antioxidants and hybrid systems remain limited, indicating a gap in comprehensive evaluation and application.

Therefore, this study aims to develop an aluminum (lignin/silica/fatty acid) hybrid (Al(LSF)) as a multifunctional bio-based additive for natural rubber (NR) composites. It is hypothesized that the incorporation of this hybrid will simultaneously enhance mechanical performance and thermo-oxidative aging resistance due to its combined organic–inorganic structure. The Al(LSF) hybrid is introduced as a multifunctional bio-based additive for NR composites. The aim is to enhance both mechanical performance and thermo-oxidative aging resistance. Dispersion behavior, interfacial interactions, curing characteristics, and mechanical properties before and after aging are systematically investigated. This work is expected to provide a sustainable strategy for designing high-performance rubber composites while contributing to the efficient valorization of agricultural waste.

## Materials and methods

### Materials

The black liquor used in this study was obtained from the alkaline solar pulping of rice straw^24,25^. Analytical-grade aluminum sulfate hexahydrate (Al₂(SO₄)₃·6 H₂O) and sodium hydroxide were purchased from Sigma-Aldrich (St. Louis, MO, USA).

Natural rubber of the SMR-20 type, density (0.913 g/cm^3^), glass transition temperature Tg = -75 °C, and Mooney viscosity ML (1 + 4) at 100 °C = 60–90, was graciously supplied by the transport and engineering company (TRENCO), Alexandria. The commercial-grade product polymerized 2, 2, 4-trimethyl-1, 2-dihydroquinoline (TMQ) as an antioxidant, stearic acid and zinc oxide (ZnO) as activators; naphthenic processing oil as a plasticizer; N-cyclohexyl-2-benzothiazole sulphenamide (CBS) as an accelerator; and elemental sulfur as a curing agent. These were provided by Aldrich Company, Germany. Sodium bentonite was provided by Alfa Aesar and Co. (Kandel, Germany). The white powder silica, commercially known as Hi-Sil 233D, was provided by PPG Industries Inc. (Delfzijl, Netherlands). Every commercial-grade rubber additive was used precisely as supplied.

**Table 1:** Composition of NR composites (phr).

Formula no./Ingredient phr	NR	NR/T_1_	NR/A_1_	NR/A_2_	NR/A_4_	NR/AA_1_	NR/AA_2_	NR/AA_4_	NR/AS_1_	NR/AS_2_	NR/AA_4_	NR/AB_1_	NR/AB_2_	NR/AB_4_
TMQ	--	1												
Antioxidant (Al (LSF) hybrid														
Al(LSF)	--	--	1	2	4	1	2	4	1	2	4	1	2	4
Antioxidant and Reinforcing filler (Al (LSF) hybrid														
Al(LSF)	--	--	--	--	--	10	15	20	--	--	--	--	--	--
Silica	--	--	--	--	--	--	--	--	10	15	20	--	--	--
Sodium bentonite	--	--	--	--	--	--	--	--	--	--	--	10	15	20

Base recipe (in phr): NR (natural rubber) 100; stearic acid 2; zinc oxide 5; CBS (N-cyclohexyl-2-benzothiazole sulfenamide) 1; processing oil 3; sulfur 3.

### Methods

#### Preparation of Al (LSF) hybrid biomass antioxidant

One liter of rice straw black liquor (pH ≈ 12) was mixed with 30 g of aluminum sulfate hydrate (Al₂(SO₄)₃·16 H₂O) under continuous stirring for 30 min at room temperature. Upon addition, the pH of the mixture decreased to approximately 4, indicating interactions between Al³⁺ ions and the functional groups of lignin and silica. The resulting precipitate was allowed to settle overnight, then separated by filtration, washed with tap water until neutral pH (≈ 7), and dried in an oven at 105 °C. This process yielded 37 g of a dark brown solid^23^.

#### Preparation of NR composites

Natural rubber (NR) composites were prepared in accordance with ASTM D3182-07(2012) using a laboratory two-roll mill of diameter 470 mm and width 300 mm, at a slow roll speed of 24 rpm and gear ratio of 1:1.4. Initially, NR was masticated at room temperature for approximately 2 min in the mixer. The compounding process was then carried out by sequential addition of ingredients under conventional mixing conditions. Zinc oxide (ZnO) and stearic acid were first incorporated and mixed for about 2 min, followed by the addition of either TMQ or the Al(LSF) hybrid at different loadings, with continuous mixing until homogeneous dispersion was achieved. Finally, CBS with sulfur was added at the final stage and mixed for additional 5 min. Different filler loadings, including silica, sodium bentonite, and the Al (LSF) hybrid, were employed as summarized in Table 1. The compounded rubbers were left overnight before vulcanization, and then the vulcanization of the composites was carried out in a compression type hydraulic press under 40 kg/cm^2^ and a temperature of 152 °C ± 1.

### Characterization

The XRF analysis elemental Analysis by wavelength dispersive X-ray fluorescence spectrometry using Axios advanced, sequential WD_XRF, spectrometer, PANalytical 2005. A JEOL JEM-2100 transmission electron microscope (TEM) (JEOL Ltd, Tokyo, Japan), equipped with an electron probe microanalyzer, was used to examine the morphology and structure of the prepared materials. The particle size was determined from TEM micrographs using image analysis software (ImageJ), where the diameters of individual particles were measured. At least 100 particles were analyzed to ensure statistical reliability.

The particle size distribution and zeta potential were further analyzed using a NICOMP 380 ZLS instrument (Particle Sizing Systems, Santa Barbara, CA, USA). The surface of rubber composites was examined using a scanning electron microscope (SEM) and energy-dispersive X-ray analysis (EDAX) utilizing a Quanta FEG 250 connected with an EDAX unit. EDAX was used to identify elements on any compound’s surface.

Rheometric characteristics of NR mixes were measured using a Monsanto oscillating disc rheometer (model 100- Akron-USA). In accordance with ASTM D2084-11 (2011), NR composites were vulcanized using an electrically heated laboratory hydraulic press at 152 °C ± 1 and cured for their optimal curing times. From them, the curing characteristics were determined as follows: M_**L**_ (dN.m): minimum torque; M_**H**_ (dN.m): maximum torque; ∆M (dN·m) torque difference, the difference between M_**H**_ and M_**L**_; t_**c90**_ (min): optimum curing time; t_**s2**_ (min): scorch time; R (dN.m/min): curing rate, defined as follows:

1 $$R = \frac{{\left( {M_{{c90}} - M_{{S2}} } \right)}}{{\left( {t_{{c90 - }} t_{{s2}} } \right)}}$$

Where M_**c90**_ (dNm) is torque at t_**c90**_ and M_**s2**_ (dN.m) is torque at t_**s2**_.

The tensile strength, stress at 100% strain, and elongation at break of the obtained vulcanizates were tested according to ASTM D412-06a (2013) with a Zwick testing machine. Three measurements were performed for each sample, and the average was taken.

The thermal aging properties were characterized as follows: after placing dumbbell-shaped specimens in a convection oven at 90 °C for an interval time of 2 and 7 days, the specimens were cooled down and kept at room temperature for at least 18 h before further tensile tests. Then, their physical properties were determined by comparing these specimens with the properties determined on the original specimens and changes noted. The determination of tensile properties and tear strength was carried out in accordance with D 412 and ASTM D 624, respectively. The thermal aging performance of the NR composites was assessed by calculating the aging coefficient (K) before and after aging using the following equations.

The aging coefficient (k) was used to evaluate the antioxidative ability of the Al(LSF) hybrid as an antioxidant for NR composites.

2 $$\:K=\frac{\left({{\upsigma\:}}_{R}\:\right)\:x\left({{\upepsilon\:}}_{R}\right)\:\mathrm{a}\mathrm{f}\mathrm{t}\mathrm{e}\mathrm{r}\:\mathrm{a}\mathrm{g}\mathrm{i}\mathrm{n}\mathrm{g}}{\left({{\upsigma\:}}_{R}\right)x\:\left({{\upepsilon\:}}_{R}\right)\:\mathrm{b}\mathrm{e}\mathrm{f}\mathrm{o}\mathrm{r}\mathrm{e}\:\mathrm{a}\mathrm{g}\mathrm{i}\mathrm{n}\mathrm{g}\:}\:\:\:\:\:\:\:\:\:\:\:\:\:\:$$

Where (σ_**R**_) is tensile strength and (ε_**R**_) is elongation at break before and after thermal aging.

The swelling characteristics and cross-link density of NR composites were evaluated in toluene as the swelling medium. The rubber vulcanizate was divided into 20 × 20 × 2 mm^**3**^ specimens, each of which was then weighed using an electronic balance. Following that, the rubber components were immersed in toluene (100 mL) for 24 h in a dark environment at room temperature. Before the rubber could be precisely weighed ($\:{W}_{0}$), the swollen piece was taken out of the solvent, and any extra solvent was removed from its surface. The following equation was used to identify the swelling’s degree:

3 $${\text{SR }} = \frac{{Ws - W0}}{{W0}}x~10$$

Where SR is the swelling ratio, Ws is the swollen weight after time t of immersion and W_0_ is the dried weight. On the basis of the equilibrium swelling state, the crosslink density was then determined using the Flory-Rehner equation^26^. The crosslink density was measured by applying the Flory-Rehner equation as given in Eqs. (4) to (6).

4 $$M_{c} = \frac{{ - \rho _{P} V_{s} V_{r}^{{1/3}} }}{{\ln \left( {1 - V_{r} } \right) + V_{r} + xV_{r}^{2} }}$$

5 $$V_{r} = \frac{1}{{1 + Q_{m} }}$$

6 $$V_{c} = \frac{1}{{2M_{c} }}$$

Where $\:{M}_{c}$ is the molecular weight between cross-links, ρ is the density of rubber for NR = 0.92 g/cm³, $\:{V}_{s\:}$is the molar volume of toluene (106.35 cm^**3**^ mol^**− 1**^), V_r_ is the volume fraction of swollen rubber.

## Results and discussion

### Characterization of Al (LSF) hybrid biomass antioxidant

#### XRF analysis

XRF analysis was performed to analysis the chemical composition of the Al(LSF) hybrid. Table 2 presents the results from the XRF scan. The data indicate that the hybrid exhibits a high LOI value (59.98%), which is contributed significantly by the high content of lignin, hence confirming that lignin forms the major content. In addition, the analysis demonstrates the presence of silica (22.76%) and aluminum oxide (Al₂O₃) (12.16%) in the Al(LSF) hybrid^27^.

**Table 2:** X-ray fluorescence (XRF) analysis of the Al(LSF) hybrid.

Main constituents	(wt%.)
SiO_2_	22.76
TiO_2_	0.01
Al_2_O_3_	12.16
Fe_2_O_3_^tot.^	0.04
MgO	0.14
CaO	0.10
Na_2_O	2.23
K_2_O	0.69
P_2_O_5_	0.11
SO_3_	1.58
Cl	0.14
LOI	59.98
MnO	0.010
NiO	0.004
CuO	0.005
ZnO	0.010
SrO	0.002
Ga2O_3_	0.003
La_2_O_3_	0.008
Br	0.005

#### Transmission Electron Microscopy (TEM), the particle size and zeta potential of Al(LSF) hybrid

**Fig. 1:**
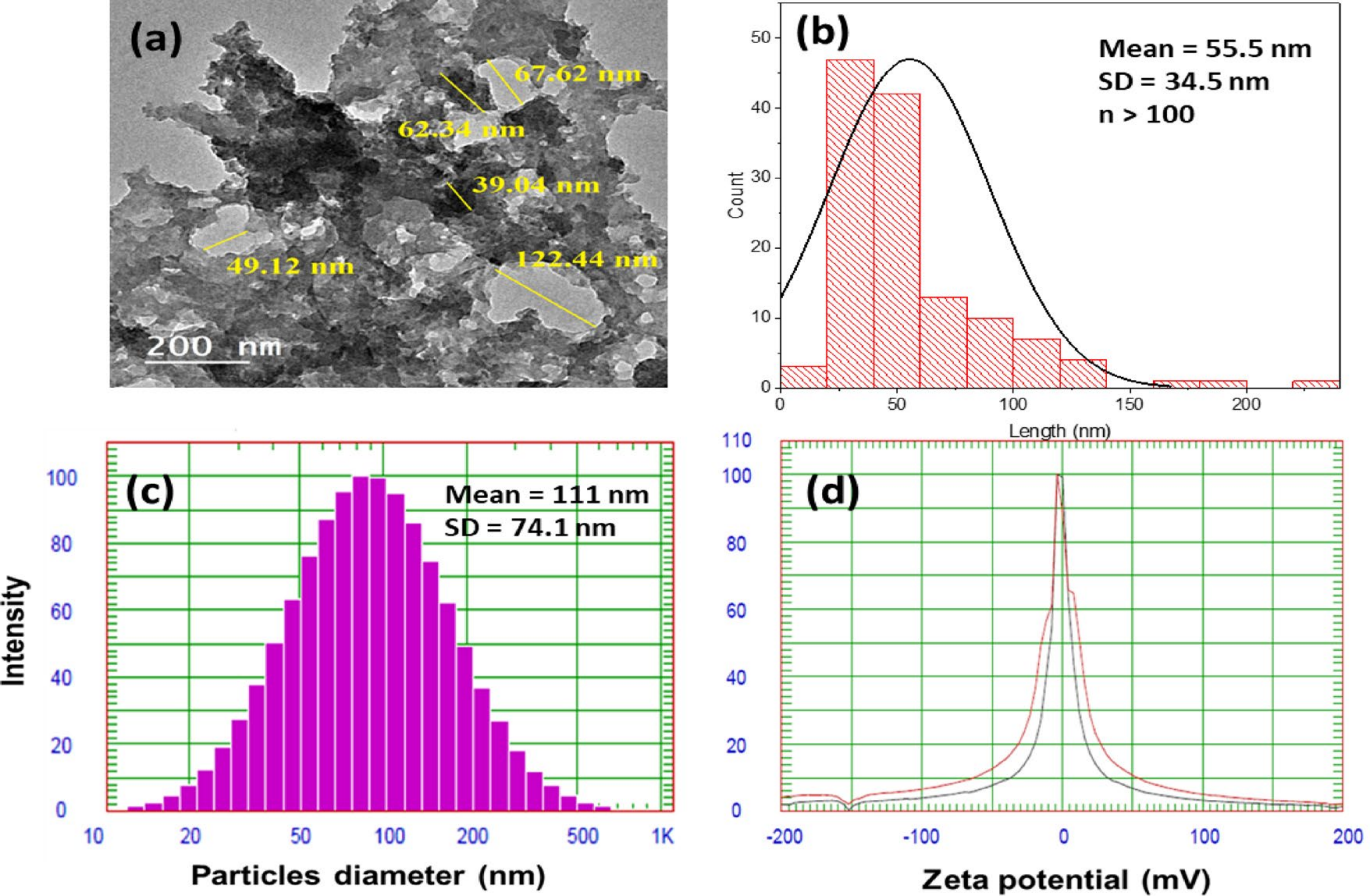
(**a**) TEM micrograph of Al(LSF) hybrid, (**b**) particle size distribution obtained from TEM image analysis (ImageJ), (**c**) particle size distribution obtained from DLS (NICOMP 380 ZLS), and (**d**) zeta potential distribution.

Size, morphology, and dispersion of the antioxidant were characterized using transmission electron microscopy (TEM). Particle size is a significant parameter that controls dispersion in the rubber matrix and consequently affects the mechanical properties of the composite. As indicated in Fig. 1a, the Al(LSF) hybrid exhibits particle sizes ranging from 39 to 122 nm. The lignin appears as dark plate-like structures, while silica appears as bright spherical particles, with partial overlap between the two phases^23^. The small particles contribute to reinforcing the rubber matrix by acting as interfacial bridging points between filler and polymer chains, enhancing dispersion and facilitating stress transfer, which improves structural integrity and uniformity.

The particle size distribution obtained from TEM image analysis is presented in Fig. 1b. The particle size was determined by analyzing at least 100 particles from different regions to ensure statistical reliability. The average particle size obtained from TEM (ImageJ analysis) was approximately 55 nm, indicating a relatively uniform distribution of particles within the hybrid system^25^. In addition, particle size distribution was further evaluated using dynamic light scattering (DLS) with a NICOMP 380 ZLS instrument, as shown in Fig. 1c, yielding a higher average particle size of approximately 111 nm. This difference is attributed to the hydrodynamic diameter measured by DLS, which includes particle agglomeration and solvation effects, whereas TEM provides the actual particle size in the dry state. This discrepancy is commonly observed between TEM and DLS measurements.

Zeta potential is widely used to evaluate the stability of nanoparticles in dispersion systems. Nanoparticles are generally considered stable when the zeta potential values are ≥ ± 30 mV due to electrostatic repulsion. As shown in Fig. 1d, the Al(LSF) hybrid exhibits a zeta potential value of − 28.80 mV, indicating moderate electrostatic stability and a low tendency toward aggregation under the intended conditions^28–31^.

#### Scanning electron microscope (SEM) of the Al(LSF) hybrid

**Fig. 2:**
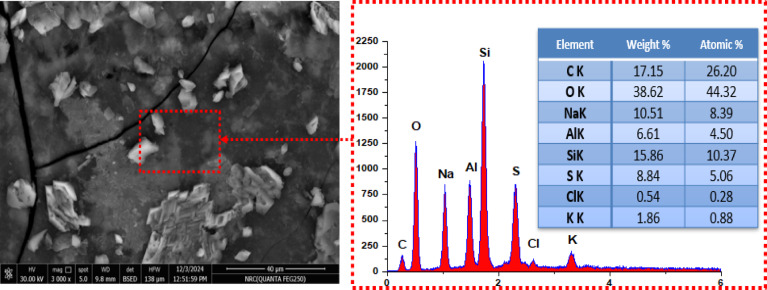
SEM image and EDS spectrum of Al(LSF) hybrid.

Figure 2 demonstrates that silica and aluminum particles are uniformly embedded within the lignin matrix. Elemental analysis via EDAX identified the following weight percentages: carbon (17.15%), attributed to lignin and fatty acids; oxygen (38.62%); aluminum (6.61%); silicon (15.86%); sulfur (8.84%); and sodium (10.51%). Furthermore, EDAX mapping revealed a consistent spatial distribution of all detected elements Al, Si, C, O, Na, and S, indicating effective integration and possible crosslinking throughout the composite structure, as presented in Fig. 2^23^.

### Characterization of NR composites

#### Morphology of NR composites

**Fig. 3:**
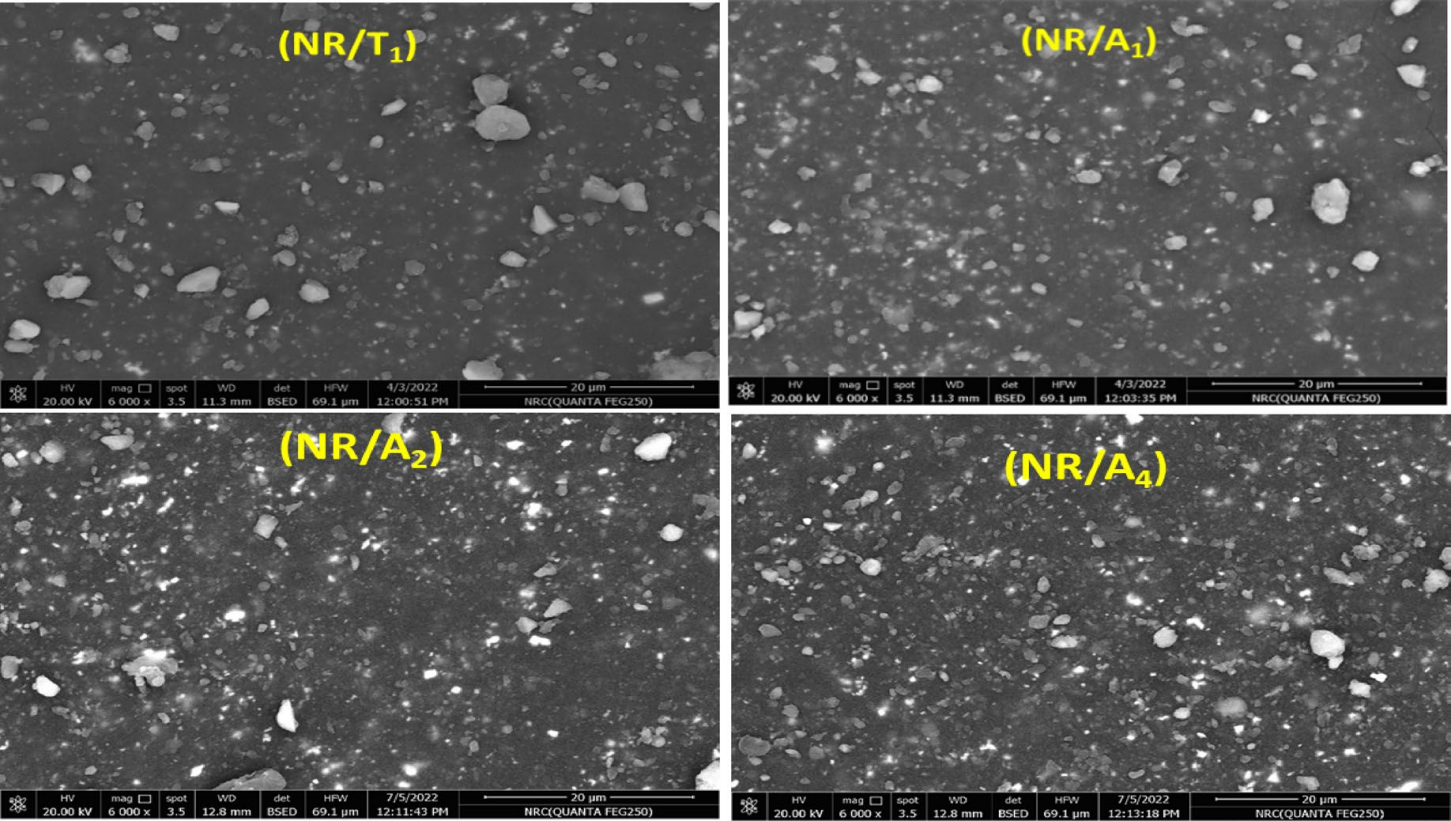
SEM micrographs of NR composites containing TMQ and NR composites incorporating the Al(LSF) hybrid antioxidant at various loadings.

The SEM micrographs of the TMQ/NR composite and Al(LSF)/NR composites reveal distinct differences in antioxidant dispersion within the NR matrix. Figure 3 shows the SEM image of NR vulcanizate containing neat TMQ and different loadings of Al (LSF) hybrid as antioxidants. The NR/TMQ composite exhibits small bright spots, and irregular features are attributed to the dispersion of the TMQ antioxidant and minor impurities inherent to the NR matrix. In contrast, replacing TMQ with the Al(LSF) hybrid leads to a pronounced reduction in domain size in the NR/A_**1**_ composite, accompanied by a more uniform distribution of finer features, reflecting improved dispersibility and compatibility of the Al(LSF) hybrid nanoparticles within the NR matrix^3^. At low Al(LSF) loading (NR/A_**1**_), the composite shows a relatively smooth surface. However, further increasing the Al(LSF) hybrid content to 2 and 4 phr results in a larger domain size and greater roughness, suggesting a deterioration in dispersion at higher loadings.

**Fig. 4:**
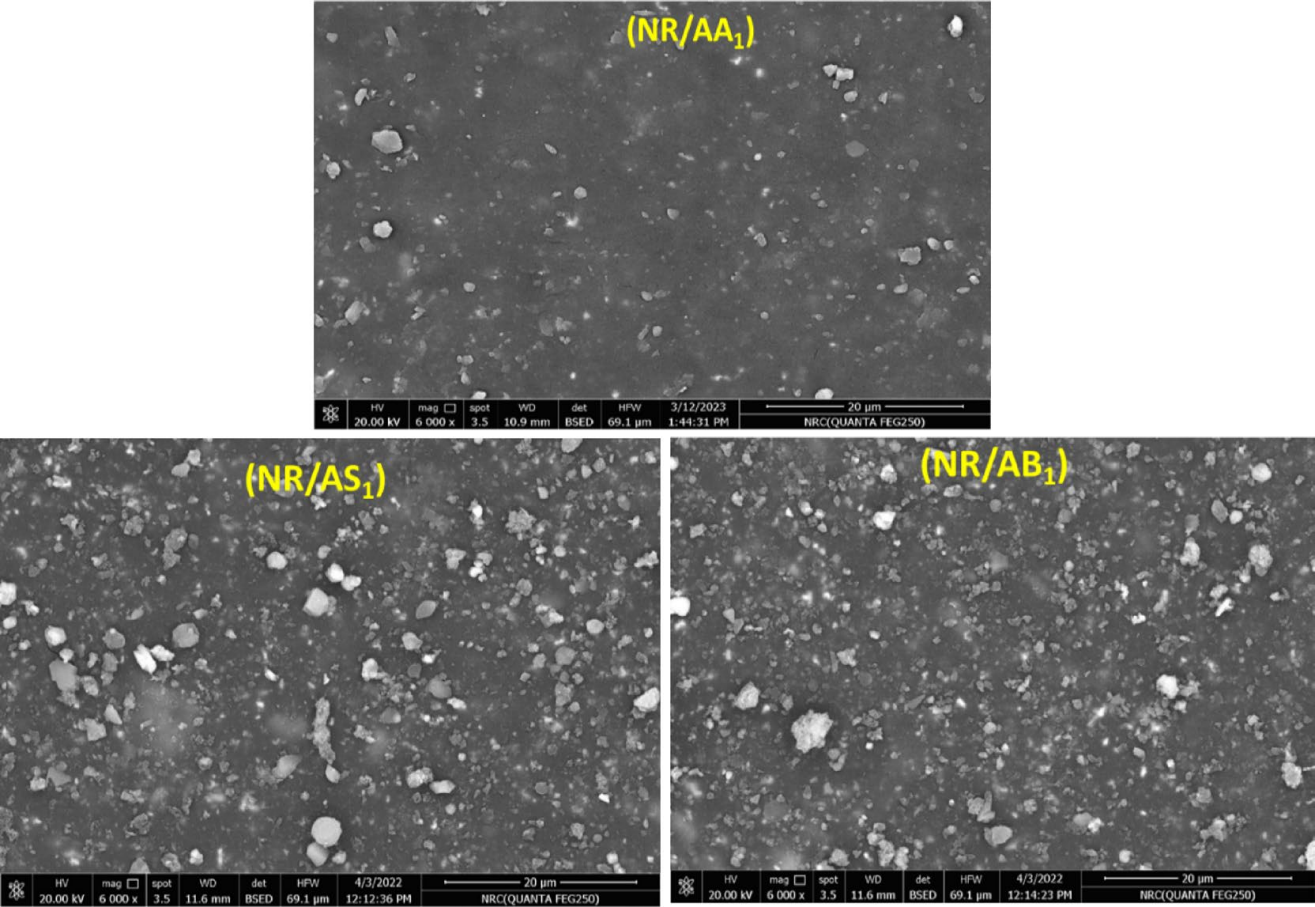
SEM images of NR composites containing 10 phr of different reinforcing fillers (Al(LSF) hybrid, silica, and sodium bentonite) in the presence of the Al(LSF) hybrid antioxidant.

The antioxidant performance of natural rubber highly depends on the dispersion of both the antioxidant and reinforcing filler within the matrix. Accordingly, the surface morphology of NR composites reinforced with Al(LSF) hybrid, silica, and sodium was examined using SEM to assess the dispersion behavior and the dual reinforcing-antioxidant role of the Al(LSF) hybrid. For the NR/AA_**1**_ composites, where Al(LSF) hybrid is used as both an antioxidant (1 phr) and a reinforcing filler (10 phr), the fracture surface is relatively smooth and homogeneous, with uniformly distributed fine features and no evident agglomeration, as shown in Fig. 4. This indicates good dispersion of the Al(LSF) hybrid within the NR matrix, which may be attributed to its nanoscale structure and the synergistic action of the silica and lignin components, which enhance interfacial interactions and compatibility with NR^23,32^. In contrast, the NR/AS**₁** composite reinforced with silica (10 phr) in the presence of Al(LSF) as an antioxidant shows a comparatively rougher fracture surface with visible particle clusters, suggesting partial agglomeration and less uniform dispersion, likely in the NR matrix due to strong filler-filler interactions^33^. Similarly, the NR/AB₁ composite reinforced with sodium bentonite (10 phr) exhibits pronounced agglomeration and a rougher morphology, which can be associated with the platelet structure and higher surface energy of bentonite, leading to limited compatibility and dispersion within the nonpolar NR matrix^34^.

#### Vulcanization properties of NR composites

**Fig. 5:**
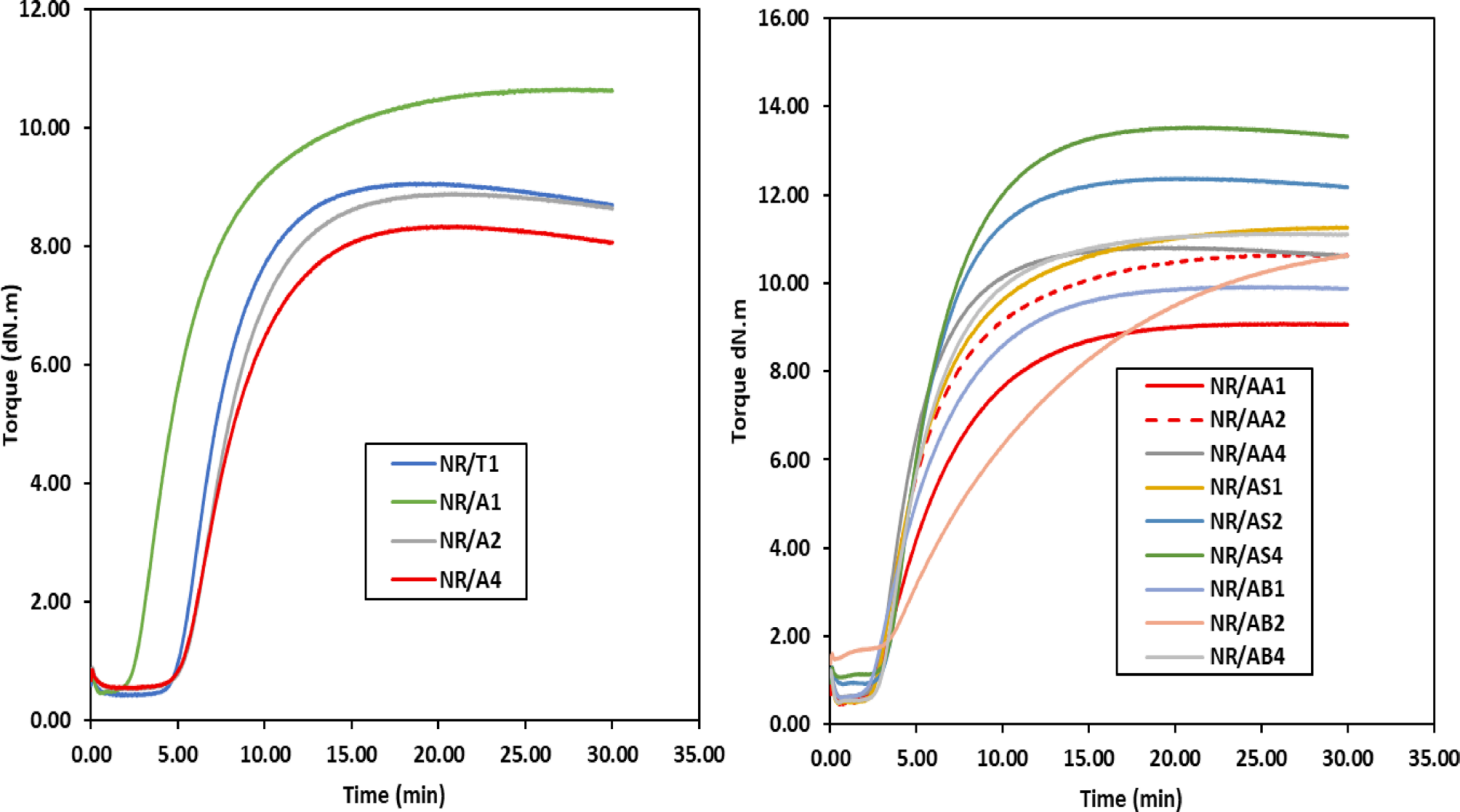
The curing curves of NR composites containing TMQ and Al(LSF) hybrid antioxidants at 152 °C.

**Fig. 6:**
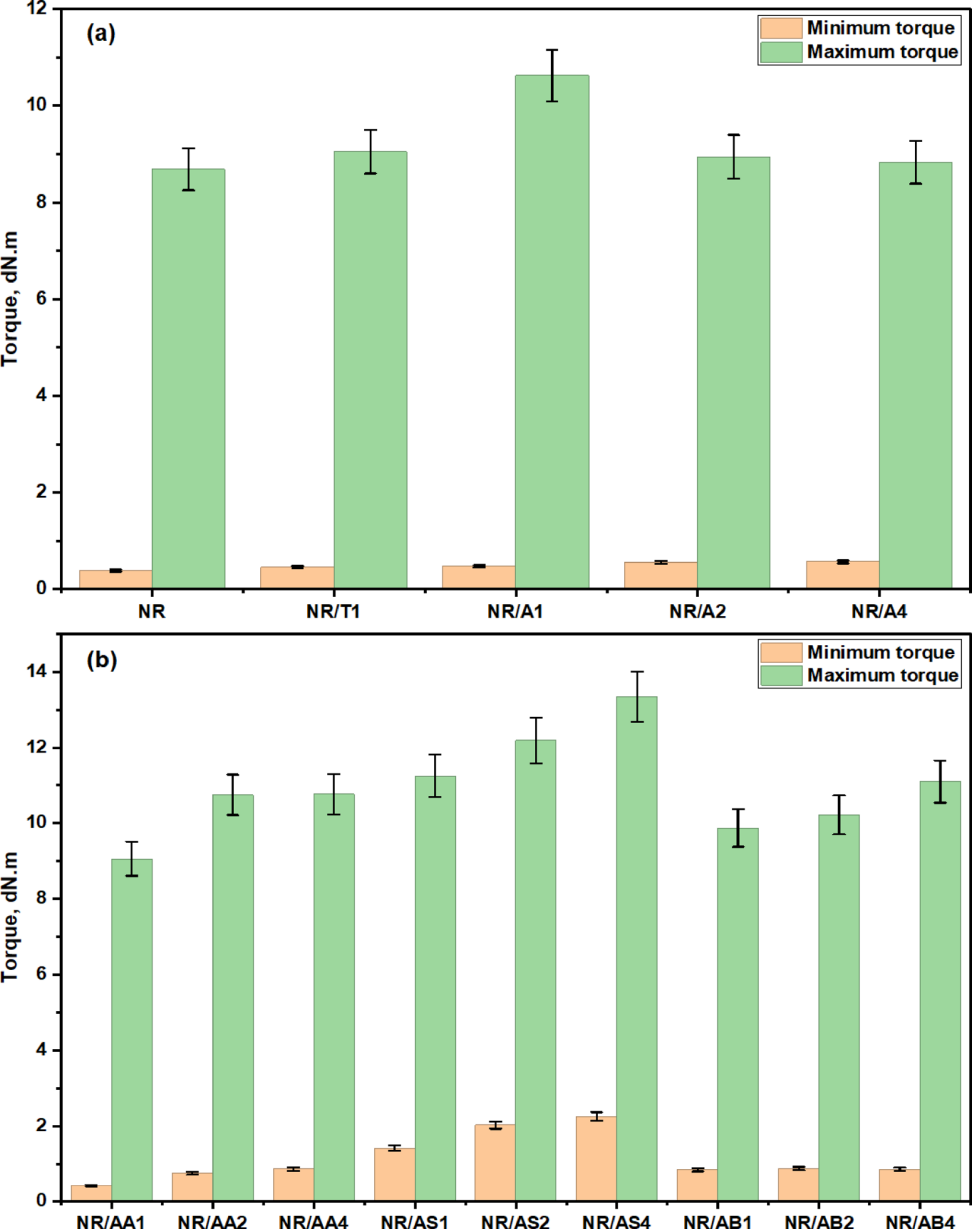
(**a**) The influence of antioxidants content on the minimum and maximum torque of the NR composites and (**b**) the minimum and maximum torque NR/fillers in the presence of Al(LSF) hybrid.

The rheometric characteristics of rubber composites play a critical role in determining the quality of rubber products intended for household applications. In this investigation, the rheometric performance of natural rubber (NR) composites incorporating either a commercial antioxidant (TMQ) or a prepared antioxidant system based on Al(LSF) hybrid was examined. Additionally, the result of varying filler loadings in the presence of Al(LSF) as an antioxidant was evaluated. These rheological properties provide essential insights into how the Al(LSF) hybrid affects curing behavior, crosslink density, and the viscoelastic characteristics of the composites, as illustrated in the corresponding figures and summarized in the accompanying tables. The torque-time curves obtained during vulcanization revealed a notable nonlinear outcome of the Al(LSF) hybrid on crosslink formation, as illustrated in Fig. 5. These rheographs depict the vulcanization behavior of NR composites filled with different concentrations of the Al(LSF) hybrid antioxidant, as well as those incorporating various types and amounts of fillers such as silica and sodium bentonite. From Fig. 6a, the NR composite without antioxidant exhibits low M_**L**_ and moderate M_**H**_, indicating enhanced processability but a comparatively lower crosslink density due to the absence of antioxidants, relative to NR composites incorporating TMQ and Al(LSF) hybrid. The cure curves for all vulcanizates containing Al(LSF) as an antioxidant exhibited similar profiles. Following the initial drop to minimum torque, the curves rose steadily to a maximum, after which they either plateaued or showed a slight decline with extended curing time, in line with previously reported observations^27^, as shown in Fig. 6a. In the case of Al(LSF)/NR composites, the maximum torque (M_**H**_) values decreased as the Al(LSF) content increased, as presented in Fig. 6. This reduction suggests weaker interfacial adhesion between the lignin component of the Al(LSF) hybrid and the NR matrix^23^. Despite this, the lignin was found to be uniformly dispersed within the rubber matrix, which contributed to improved scorch resistance and extended optimum cure time. Among the formulations, the composite containing 1 phr of Al(LSF) (NR/A_**1**_) exhibited the most favorable properties. It was observed that incorporating Al(LSF) into NR compounds led to a reduction in M_**H**_, while the minimum torque (M_**L**_) showed a slight increase with higher Al(LSF) loadings. The rise in M_**L**_ indicates increased compound viscosity and stronger interactions among antioxidant molecules^23,35^. This increase in minimum torque is likely due to the formation of premature crosslinks during the mixing process.

Further analysis of the rheometric data for NR composites reinforced with Al(LSF), silica, and sodium bentonite presented in Tables 3 and 4 and the corresponding figures revealed that the inclusion of these fillers, in the presence of Al(LSF) as an antioxidant, resulted in increased M_L_ and M_**H**_ values, as well as prolonged optimum cure time. The increase in M_**L**_ reflects a rise in viscosity prior to the onset of vulcanization, while the increase in M_**H**_ indicates enhanced viscosity and greater crosslink density in the cured composites. The higher torque values observed at elevated filler loadings may be attributed to interactions between the filler and the curing agents, leading to a modest increase in crosslink density. Additionally, these increments could stem from physical interactions between the NR matrix and the filler surfaces, as well as hydrodynamic effects^23,36^. The maximum torque (M_**H**_) is often interpreted as an indicator of the composite’s modulus^37^, with higher values suggesting increased resistance to flow due to the presence of fillers^38^. Compared to Al(LSF)/NR composites, those reinforced with sodium bentonite or commercial silica exhibited slightly higher torque values^37^. This increase points to stronger physical crosslinking between these fillers and the NR matrix, likely resulting from the formation of filler-rubber linkages and a higher cross-link density in the Al(LSF)/NR composites.

**Fig. 7:**
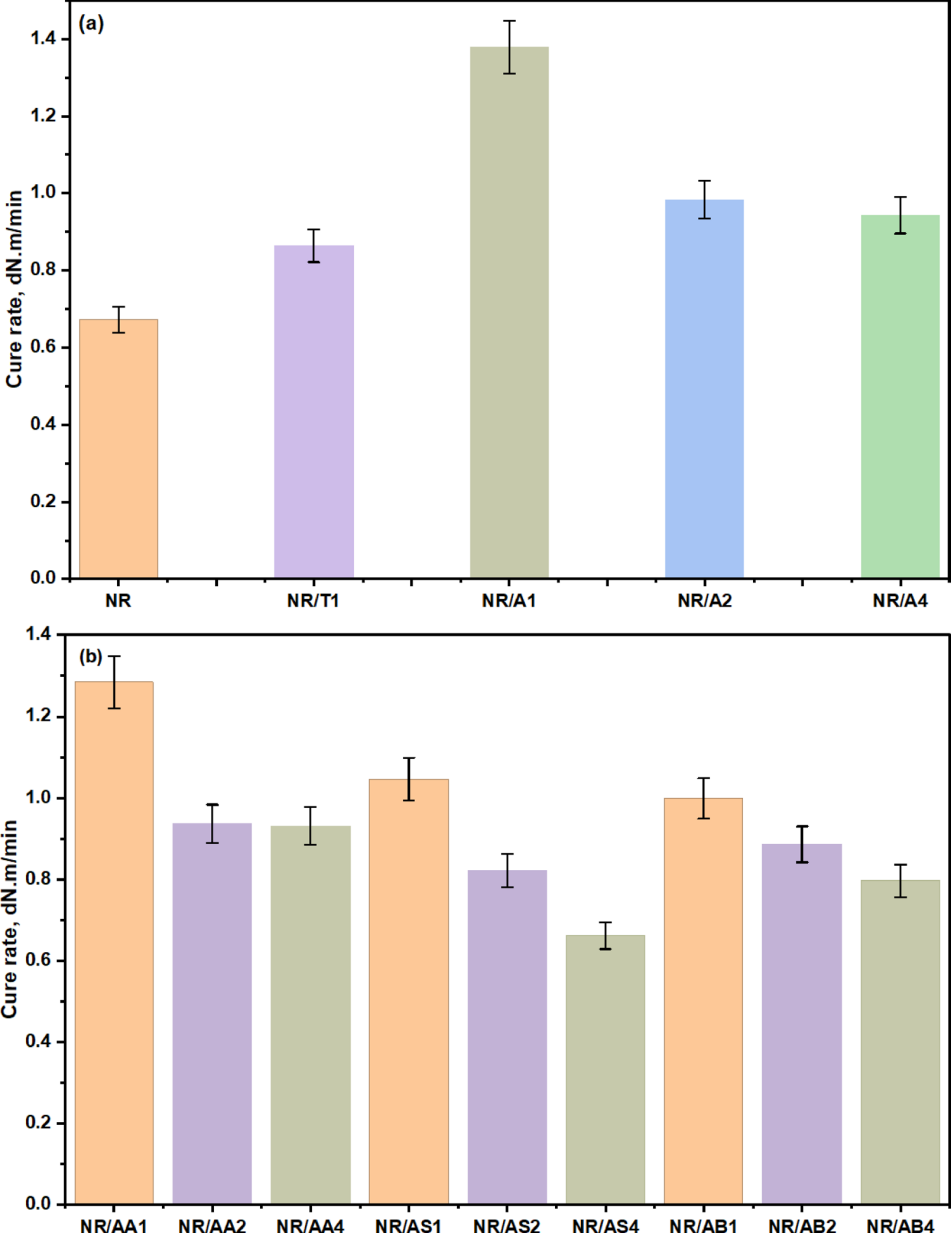
(**a**) The influence of antioxidants content on the cure rate of the NR composites and (**b**) the cure rate of NR/fillers in the presence of Al(LSF) hybrid.

In most cases, the t_**c90**_ was significantly prolonged at the highest antioxidant content (from Table 3, NR/A_**4**_ (4 phr)) or the highest filler content (Table 4: NR/AA_**2**_ (15 phr), NR/AS_**4**_ (20 phr), and NR/AB_**4**_ (20 phr). The kinetics of the curing process were further examined by evaluating the cure rate R. As illustrated in Fig. 7, it is evident that the highest cure rate was observed for the rubber compounds NR/A_**1**_ (1 phr antioxidant) and NR/AA_**1**_ (10 phr filler). Increasing the amount of antioxidant or filler resulted in a lower cure rate. Regarding the type of filler, the compound filled with (Al (LSF) hybrid biomass (10 phr) exhibited the highest cure rate. The most substantial reduction in cure rate occurred at high filler loadings. Moreover, as filler content increased, the differences in cure rate became less pronounced. This decline in curing kinetics may be attributed to the polarity of certain fillers, such as silica, which can absorb or dilute the curing system additives, thereby reducing their effectiveness during vulcanization^39^. In conclusion, a high R value, as seen in NR/AA_**1**_, indicates a fast-curing rubber (CRI = 16.18) with rapid crosslink development advantageous for productivity but potentially problematic if not properly controlled.

**Table 3:** Rheometric characteristics of NR/antioxidant composites at 152 °C.

Sample code	NR	NR/T1	NR/A1	NR/A2	NR/A4
Torque difference, dNm	8.3	8.59	10.15	8.39	8.26
Scorch time (Ts2), min	2.82	3.33	3.36	3.57	4.44
Optimum cure time (T_C90_), min	13.7	12.05	9.84	11.08	12.16
CRI, min^− 1^	9.19	11.47	15.43	13.32	12.95
ὴ_r_	--	1.18	1.23	1.436	1.462
S_r_	--	1.041	1.22	1.029	1.016
σ_anti_	--	3.494	22.289	0.542	-0.1205

**Table 4:** Rheometric characteristics of NR/filler composites in the presence of Al(LSF) as antioxidant at 152 °C.

Sample code	NR/AA_1_	NR/AA_2_	NR/AA_4_	NR/AS_1_	NR/AS_2_	NR/A_S4_	NR/AB_1_	NR/AB_2_	NR/AB_4_
Torque difference, dN.m	8.64	10	9.91	9.84	10.17	11.09	9.03	9.35	10.25
Scorch time (T_s2_), min	5.84	3.72	3.56	3.89	4.38	4.36	3.3	3.2	3.23
Optimum cure tim (T_C90_), min	11.76	13.84	13.03	12.17	15.28	19.11	11.96	12.49	14.56
CRI, min^− 1^	16.18	9.88	10.56	12.08	9.17	6.78	11.55	10.76	8.83
ὴ_r_	1.077	1.923	2.205	3.615	5.179	5.769	2.154	2.231	2.179
S_r_	1.046	1.237	1.239	1.295	1.403	1.531	1.136	1.199	1.951
α_anti_	4.096	10.241	4.849	18.554	11.265	8.795	8.795	6.795	5.874

α_**anti**_ is specific constant for reinforcement of antioxidant or fillers (i.e., activity of antioxidant or filler); ὴ_r_ is the relative viscosity; $\:{\mathrm{S}}_{\mathrm{r}}\:\mathrm{i}\mathrm{s}\:$ the relative modulus, where phr is part per hundred parts of rubber.

#### Reinforcing efficiency of Al (LSF) hybrid as antioxidant for NR composites

The degree of reinforcement of antioxidants of NR rubber composites in the presence of aromatic antioxidants (TMQ) or prepared Al (LSF) hybrid was calculated by Lee^39^ and other researchers^40–47^. Calculated the reinforcing efficiency of plasticity ($\:{\alpha\:}_{anti}$.) as given by the following equation:

7 $$\frac{{(M^{\prime}_{H} - ~M^{\prime}_{L} ~)_{{anti}} }}{{(M_{H}^{0} - M_{L}^{0} )_{{gum}} }} - 1 = \alpha _{{anti}} ~~\left( {\frac{{m_{{anti}} }}{{m_{{gum}} }}} \right)$$

Where $(M^{\prime}_{H} - ~M^{\prime}_{L} )_{{anti}}$ are the changes in torque that occur during vulcanization of the tested composites in the presence of antioxidants, $\:({\mathrm{M}}_{\mathrm{H}}^{0}-{\mathrm{M}}_{\mathrm{L}}^{0}{)}_{\mathrm{g}\mathrm{u}\mathrm{m}}$ is the changes in torque without antioxidant, and $\:{{\upalpha\:}}_{\mathrm{a}\mathrm{n}\mathrm{t}\mathrm{i}}$ is a factor representing the reinforcement factor or the rubber–antioxidant interaction, and it is independent of the cure system and strictly related to the antioxidant morphology. Table 3 shows the α_**anti**_ values of natural rubber (NR) combined with various antioxidants. Among the formulations, the highest α-anti was observed with the addition of 1 phr of Al(LSF) hybrid, surpassing both other loadings of the same filler and those containing TMQ. This increase indicates stronger interfacial interaction between the NR matrix and the Al(LSF) hybrid. Further analysis reveals that incorporating different fillers, namely Al(LSF) hybrid, silica, and sodium bentonite, at varying concentrations (10, 15, and 20 phr) resulted in elevated α-anti values for NR composites containing the prepared antioxidant (NR/AA_**2**_). Specifically, NR compounded with 10 phr silica (NR/AS_**1**_), 15 phr silica (NR/AS_2_) and 10 phr sodium bentonite (NR/AB_1_) also exhibited notably higher α-anti values. These improvements are attributed to better filler dispersion and enhanced NR–filler adhesion facilitated by the antioxidant. This interpretation is consistent with the findings presented in Table 4. The degree of Al (LSF) hybrid dispersion in the NR rubber matrix can be determined quantitatively by the following equations:

8 $$L{\text{ }} = \mathop \eta \limits^{'} \mathop r\limits^{'} - {\text{ }}Sr$$

Where

$$\mathop \eta \limits^{'} \mathop r\limits^{'} = ~\frac{{D_{{\min }}^{f} }}{{D_{{\min }}^{0} }}\,\,\,\,\,\,\,\,\,\,\,\,\,\,\,\,\,\,\,\,S_{r} = ~\frac{{D_{{\max }}^{f} }}{{D_{{\max }}^{0} }}$$

As well, Lee assumed that the relative viscosity ($\mathop \eta \limits^{'} \mathop r\limits^{'}$), is higher than the relative modulus ($\:{S}_{r}$) i.e. $\mathop \eta \limits^{'} \mathop r\limits^{'} > S_{r}$.

**Fig. 8:**
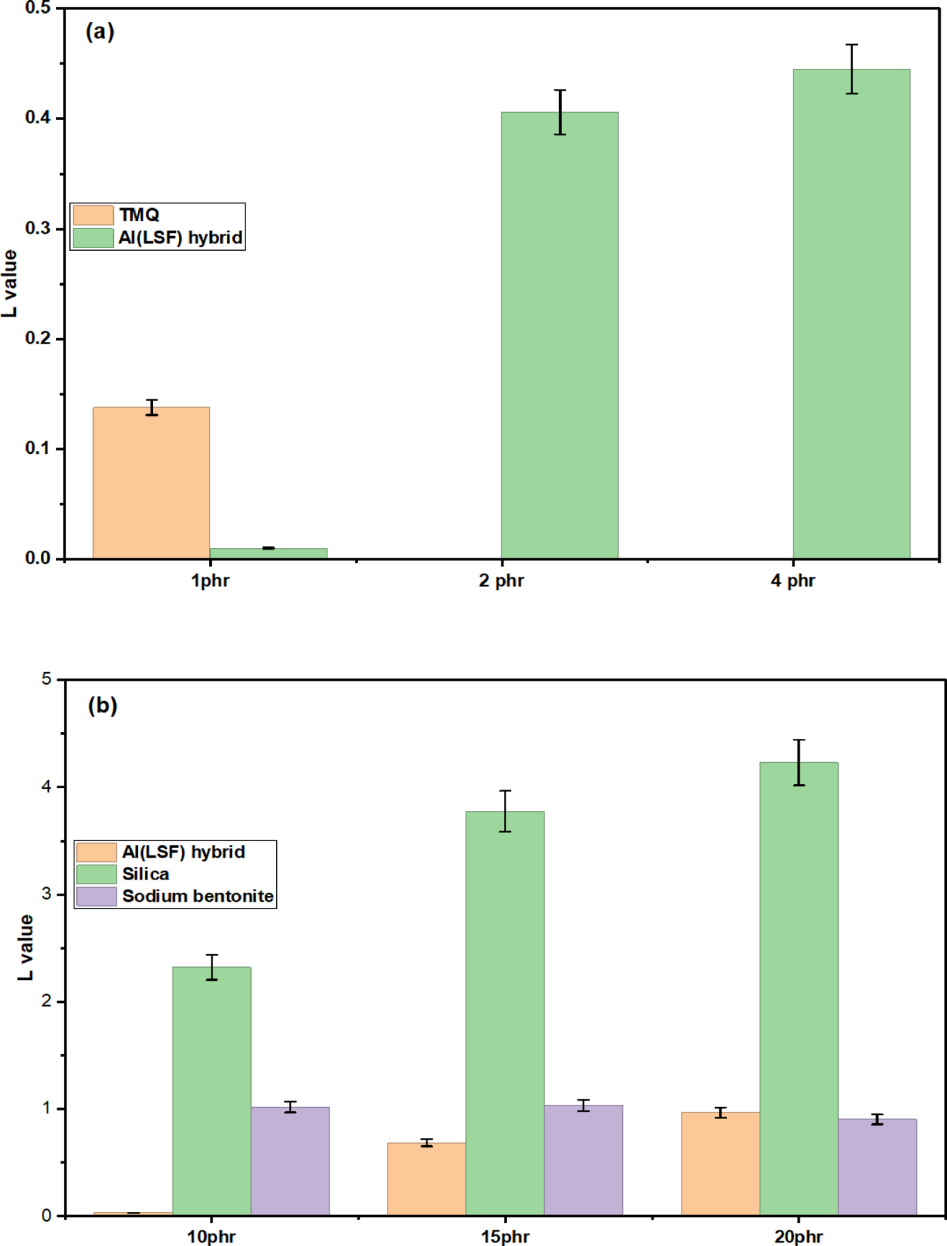
Relation between the L value of NR composites and (**a**) antioxidant content and (**b**) different filler types in the presence of the Al(LSF) hybrid as antioxidant.

Tables 3 & 4 and Fig. 8 exhibit the calculated $\mathop \eta \limits^{'} \mathop r\limits^{'} ,S_{r} {\mathrm{~~}}and{\mathrm{~}}$L values, which are $\mathop \eta \limits^{'} \mathop r\limits^{'}$the relative viscosity and $\:{S}_{r}$ the relative modulus. Tables 3 and 4 present the calculated values of ῆr’ and Mr, while the corresponding L values are illustrated in Fig. 8. It is evident that in natural rubber (NR) composites containing either TMQ or Al(LSF) as antioxidants, along with various fillers, the filler particles are well dispersed. Specifically, improved dispersion is observed in NR compounded with 10 phr Al(LSF), NR with 20 phr sodium bentonite, and NR with 10 phr silica loading. A lower L value indicates better dispersion of fillers within the NR matrix. Both ῆr’ and Sr values increased with higher filler loadings, suggesting an enhancement in the relative viscosity and relative modulus of the elastomer. However, at 20 phr Al(LSF) loading, a decrease in Sr was observed. Additionally, L values decreased with improved dispersion, reinforcing the inverse relationship between L and filler dispersion quality.

At higher filler loadings, particularly in NR/AA_**4**_, NR/AS_**4**_, and NR/AB_**2**_, a greater divergence between ῆr’ and Sr values was noted. Meanwhile, the increase in L with filler loading suggests the onset of filler agglomeration within the NR matrix^40–47^. These observations are consistent with the data presented in Tables 3 and 4 and the corresponding Fig. 8.

### Thermo-oxidative of NR composites

#### Mechanical performances of NR composites before and after aging process

**Fig. 9:**
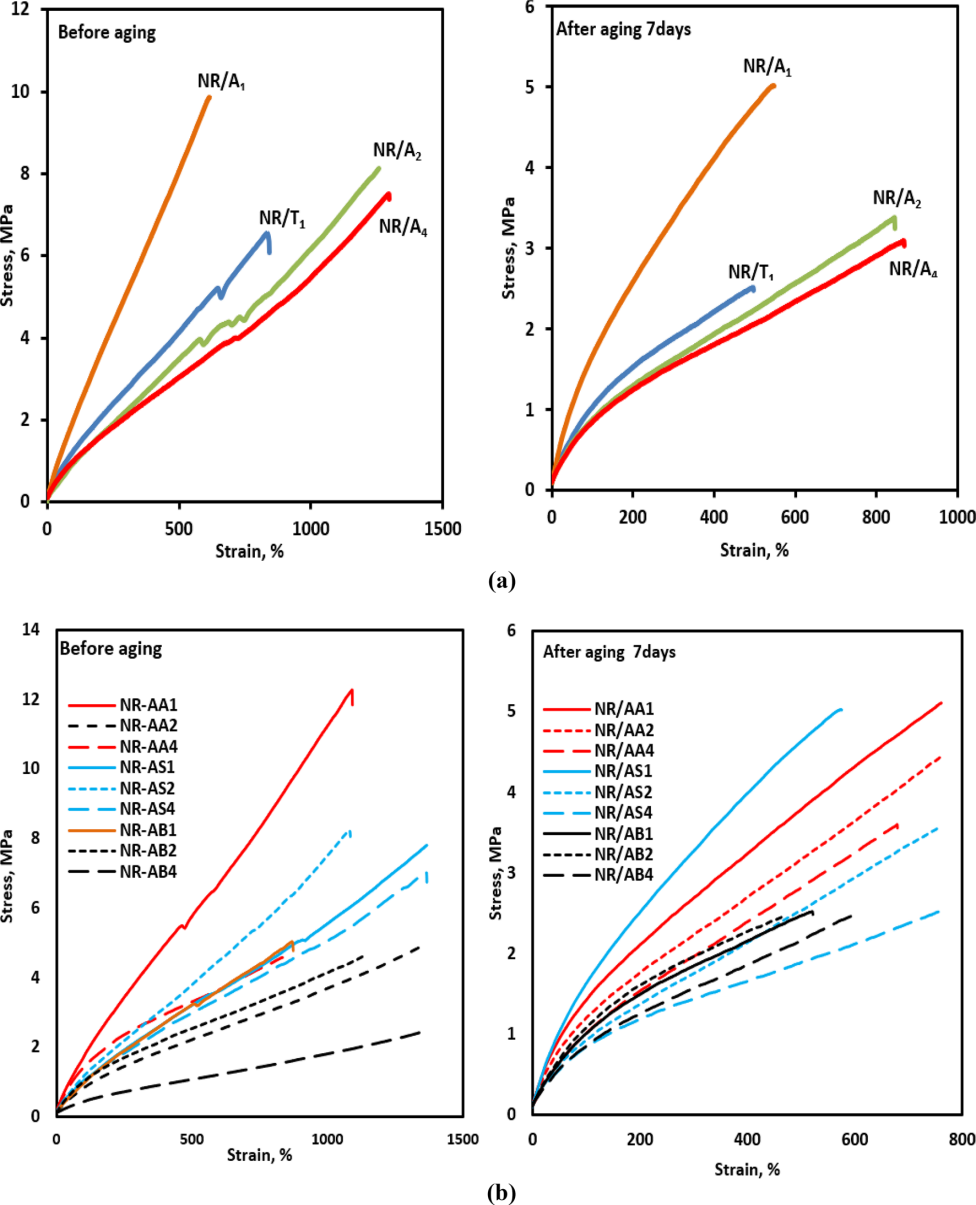
(**a**) Stress-strain curves of NR composites containing TMQ and Al(LSF) hybrid antioxidants: before and after aging 7 days. (**b**) Stress-strain curves of NR composites with different fillers in the presence of the Al(LSF) hybrid antioxidant: before and after aging 7 days.

The aging process of natural rubber (NR) is driven by exposure to oxygen, heat, and mechanical stress during processing, storage, and use. Owing to its unsaturated polymer backbone, NR is highly susceptible to oxidative degradation, which causes polymer chains scission and reduces molecular weight and chain entanglement. Ultimately deteriorates mechanical properties^48^. Figure 9a illustrates stress-strain curves of NR composites containing TMQ and different loadings of the Al(LSF) hybrid antioxidant before and after aging, indicating enhanced tensile performance and aging stability at low Al(LSF) content (1 phr). In addition, as shown in Fig. 9b, NR composites containing Al(LSF) act as both an antioxidant and a reinforcing filler at low loading, exhibiting better performance than composites filled with silica or sodium bentonite.

**Fig. 10:**
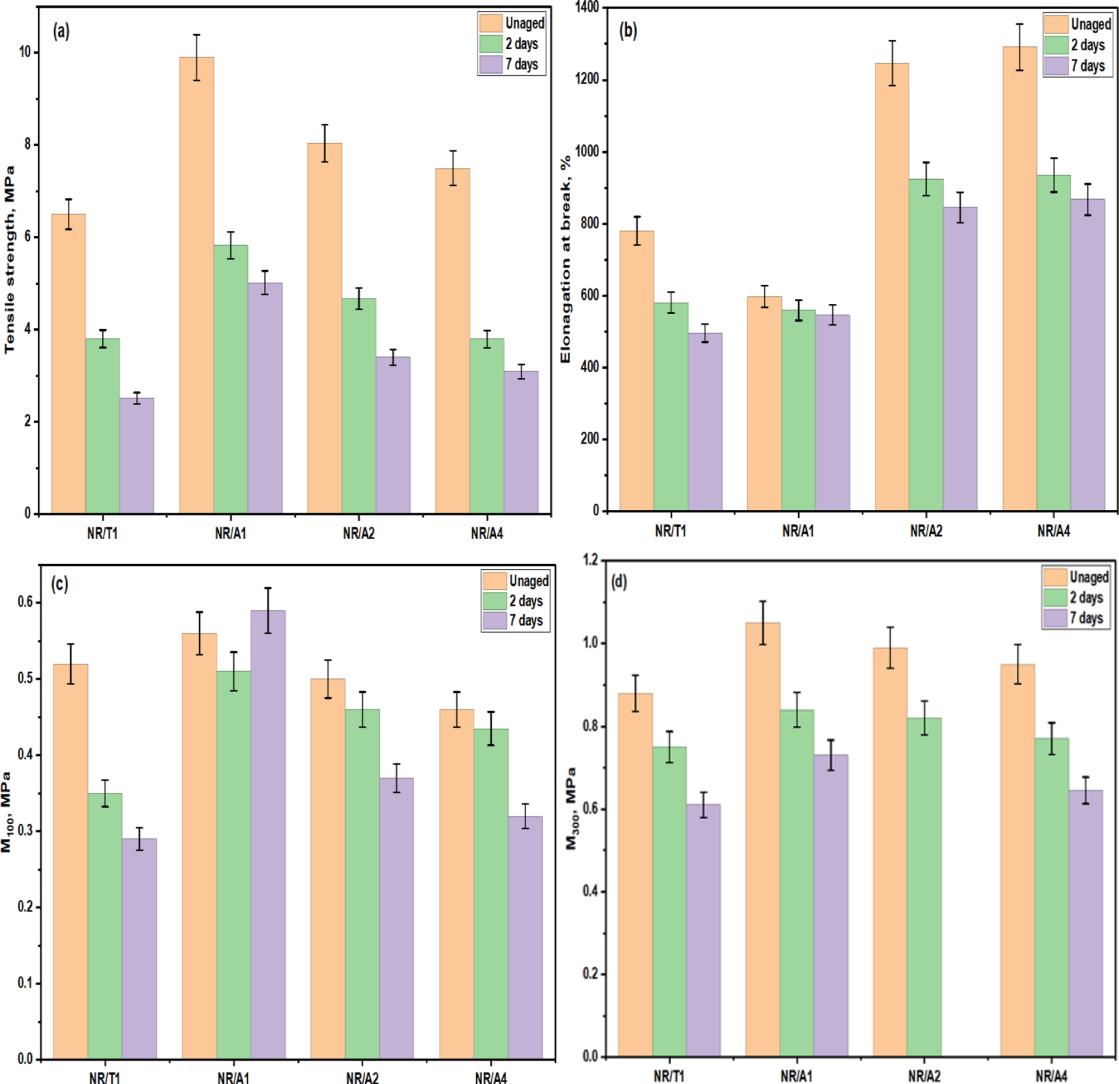
Mechanical properties of NR composites before and after aging: (**a**) tensile strength, (**b**) elongation at break, (**c**) modulus at 100% strain, and (**d**) modulus at 300% strain.

Mechanical properties are essential for the practical utilization of rubber products. However, thermal and oxidative exposure enhances the crosslinking density of rubber, resulting in the deterioration of the mechanical properties of the NR composite materials. Tensile properties of vulcanizates with different Al(LSF) hybrid loadings are compared with those of the commercial antioxidant TMQ. According to Fig. 10, NR/TMQ composites exhibited a pronounced reduction in tensile strength and elongation at break after 2 and 7 days of aging, whereas NR/Al(LSF) composites showed a relatively slow change. The modulus values at 100 and 300% elongation, denoted as M_**100**_ and M_**300**_, represent the tensile stress at the corresponding strain^37^. As shown in Fig. 10c,d, M_**100**_ values of NR/Al(LSF) composites are higher than those of NR/TMQ composites, and a similar trend is observed for M_**300**_. Relative to TMQ, the addition of Al(LSF) hybrid could more effectively retard the thermal oxidation aging of the NR vulcanizates^49,50^.

**Fig. 11:**
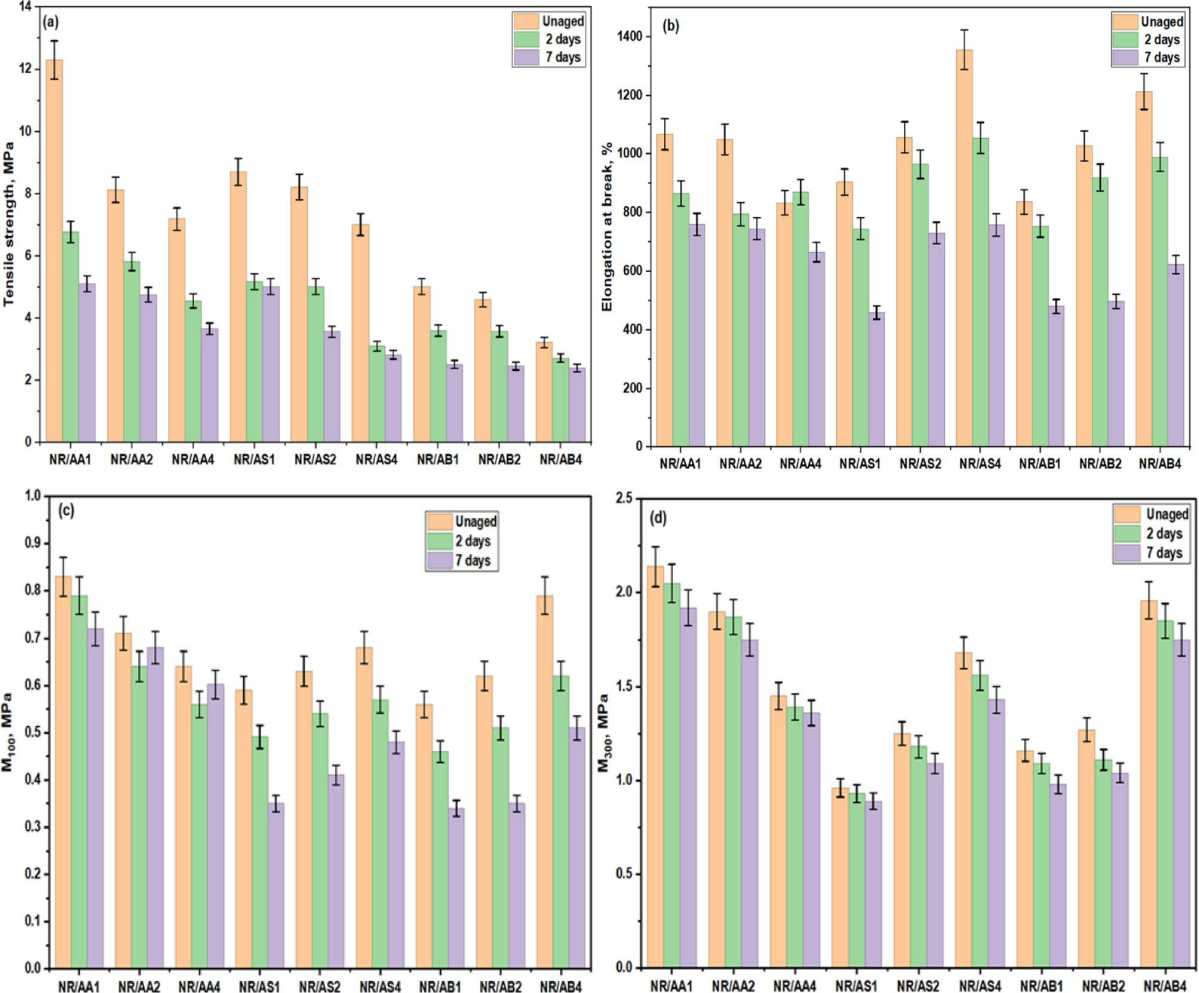
Mechanical properties of NR composites with different fillers in presence Al(LSF) hybrid antioxidant before and after aging: (**a**) tensile strength, (**b**) elongation at break, (**c**) modulus at 100% strain, and (**d**) modulus at 300% strain.

The influence of different loadings of the Al(LSF) hybrid antioxidant on the mechanical properties of NR composites containing different fillers Al(LSF) hybrid, silica and sodium bentonite) as function of aging time. As shown in Fig. 11a, NR composites containing the Al(LSF) hybrid exhibit superior tensile strength (12.3 MPa) compared with those filled with silica or sodium bentonite, especially at low Al(LSF) loading when used as either an antioxidant or reinforcing filler before aging. This enhancement is attributed to the presence silica and lignin Al(LSF) hybrid, which provides effective reinforcement, uniform dispersion, and strong interfacial interactions between the filler and NR phases^51^. However, increasing the loading of the Al(LSF) antioxidant and fillers Al(LSF), silica, and sodium bentonite) leads to a gradual reduction in tensile strength suggesting that excessive filler content restricts chain mobility and reduces reinforcement efficiency due to filler-filler interactions or agglomeration. All NR composites presented a dramatic reduction in tensile strength after aging for 3 and 7 days, but NR composites filled with Al(LSF) showed a relatively slow change. This indicates that Al(LSF) acts as an effective antioxidant and filler, providing short-term thermo-oxidative aging resistance, which can be attributed to the hindered phenolic hydroxyl groups in lignin that inhibit the reactions induced by oxygen and its radical species^52,53^.

Meanwhile Fig. 11b shows that elongation at break of NR composites filled with Al(LSF) hybrid decreases slightly with increasing filler loading, whereas composites filled with silica or sodium bentonite exhibit increased elongation at break as the filler content increases in presence of Al(LSF) hybrid as antioxidant. This behavior can be attributed to excessive Al(LSF) hybrid addition, which increases vulcanizate network rigidity and restricted polymer chain mobility leading to a reduction in crosslink densities of NR vulcanizates. In contrast, the higher elongation observed in the other NR composites is associated with the plasticization complex interaction results from plasticization effects that interfere with vulcanization and promote chain extensibility flexibility^48^. These results are in good agreement with the rheometric properties.

For all NR vulcanizates, elongation at break decreased after 2 days of aging and further progressive reduction as the aging time increased to 7 days. However, the rate of decrease showed no significant among the NR composites with the varying fillers content. In addition, Fig. 11c,d show the modulus at 100% and 300% elongation of the NR decreased with increasing Al(LSF) hybrid content. Notably, NR composites with Al(LSF) hybrid exhibited higher modulus values compared with other NR composites, while the change in modulus after aging was lower than that observed for silica and bentonite filled composites. Stable modulus values indicate high resistance to aging effects. 

**Fig. 12:**
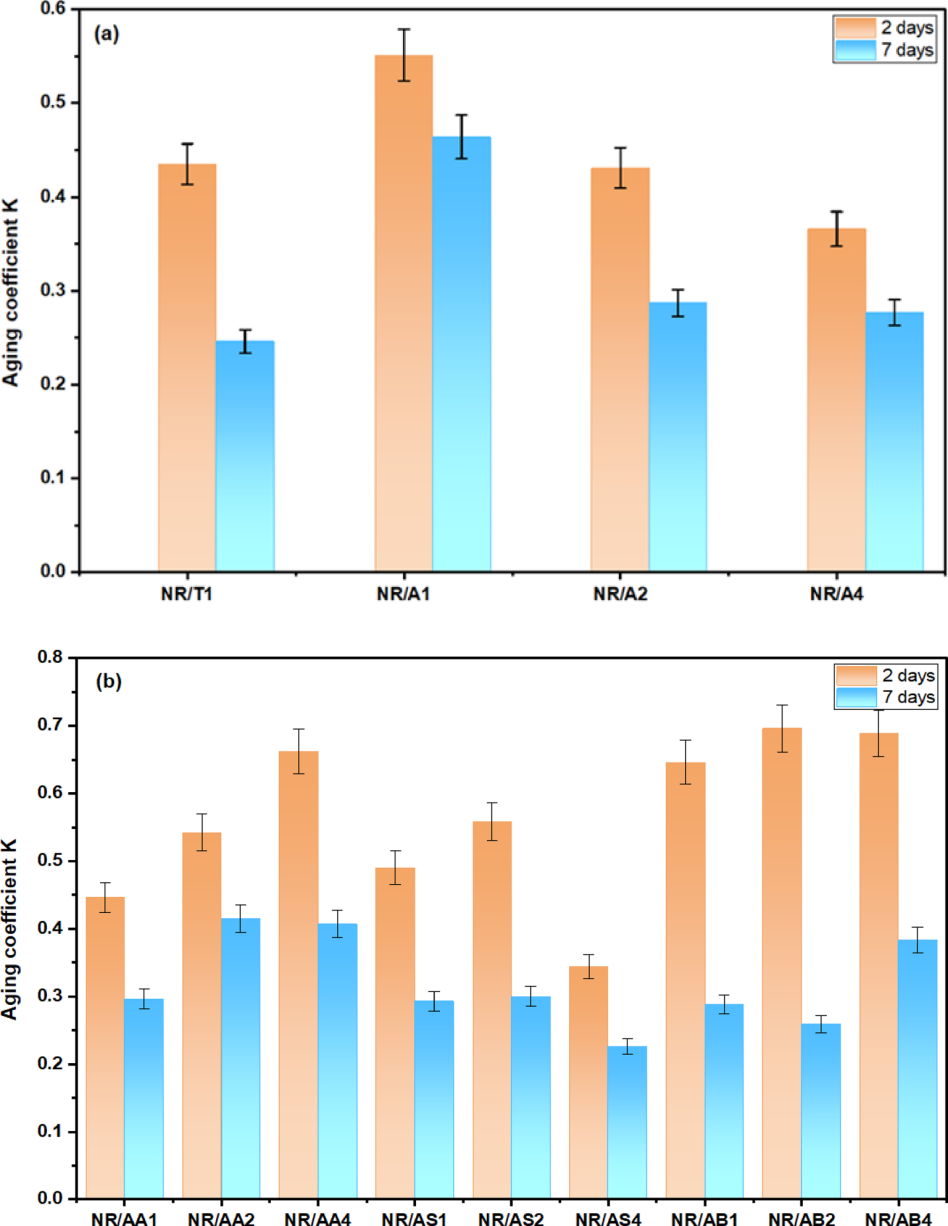
Aging coefficient (K) of NR composites before and after thermal aging at different aging times (2 and 7 days).

The aging coefficient (K) serves as an overall indicator of the thermo-oxidative aging resistance of rubber materials. As shown in Fig. 12a, NR/A_**1**_ composites containing 1phr Al (LSF)) exhibited the highest K value compared to TMQ and other Al (LSF) loadings under identical aging conditions, reflecting its superior stability. During thermal aging, they are continuously generated after the initial stage. However, the low loading of the Al (LSF) hybrid effectively limited antioxidant migration, resulting in a long-term antioxidant effect^48,53^. As presented in Fig. 12b, the NR composites incorporating the Al (LSF) hybrid as both antioxidant and filler exhibit substantially enhanced anti-aging performances. In contrast, increasing the loading of the Al(LSF) hybrid antioxidant resulted in a significant enhancement of the K value in Al (LSF)/NR and sodium bentonite/NR composites relative to the silica/NR composite. Notably, Al (LSF)/NR composites maintain substantially higher K values than the other formulations after aging 7 days. The ability to retain a high aging coefficient value during long-term high-temperature conditions indicates the excellent thermo-oxidative aging resistance of the Al (LSF) hybrid. This finding is critical for maintaining NBR performance in long-term high-temperature applications^54^. Overall, thermal aging tests confirmed that Al (LSF) hybrid is an effective antioxidant for the elastomer by scavenging oxygen radicals and suppressing oxidative chain reactions.

#### Crosslinking density measurements

**Fig. 13:**
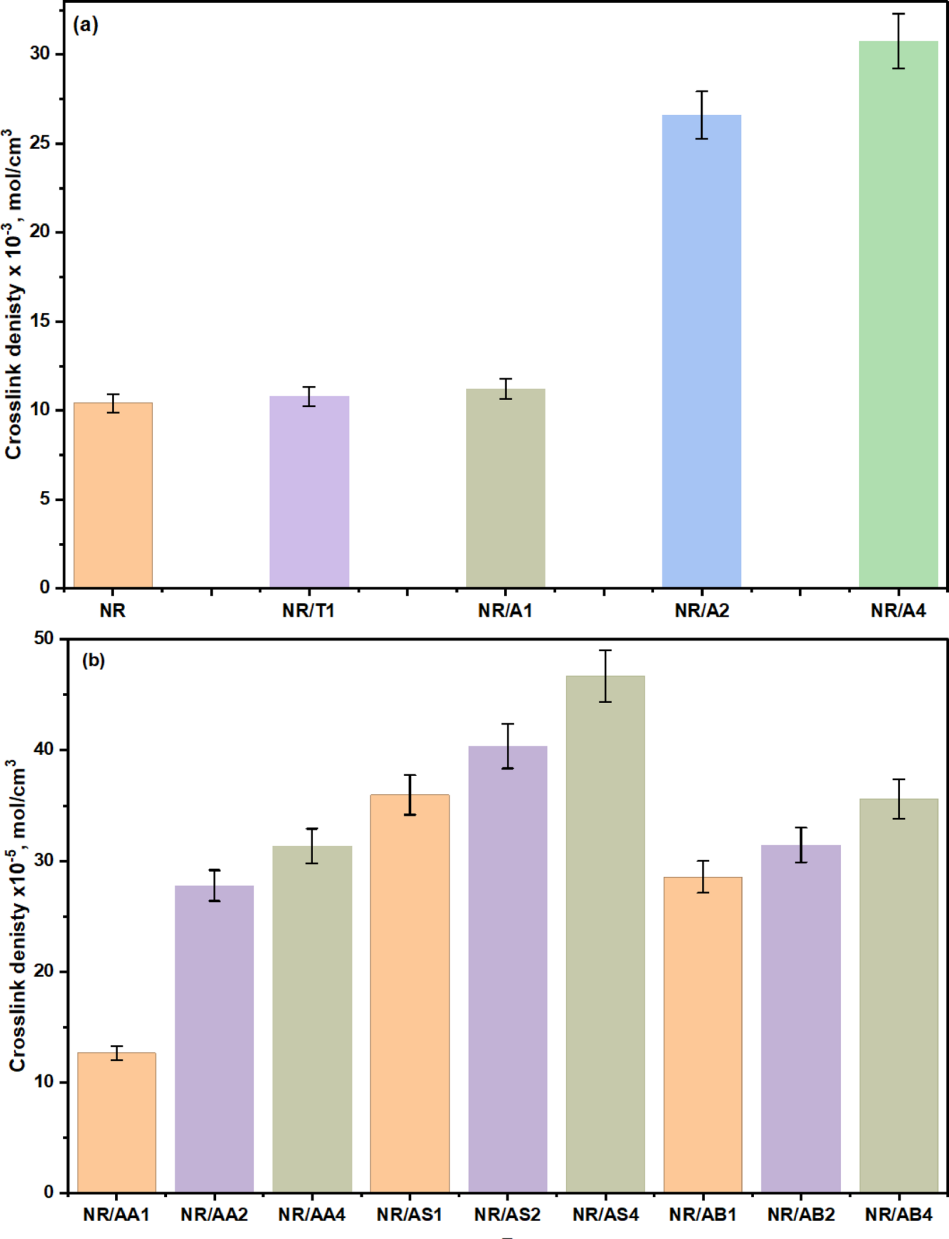
(**a**) The influence of commercial and prepared antioxidants content on the crosslink density the NR composites and (**b**) the crosslink density of NR/fillers in the presence of Al(LSF) hybrid.

From the Flory-Rehner relation equation’s calculation of the crosslink density, it was observed that it increases with increasing the content of Al(LSF). This refers to the presence of Al(LSF) as an antioxidant in the composite, which causes the extra physical and chemical cross-links in the NR composites. This result confirms with the data in Fig. 13 shows the crosslinking density of natural rubber, unfilled and filled with commercial, prepared antioxidants, and the investigated fillers as determined by the immersion method. The crosslinking density of NR composites increased as the content of prepared antioxidants Al(LSF) or investigated filler in the presence of Al(LSF) as an antioxidant increased in the system. The crosslinking density is clearly higher for NR composites, including 4 phr of Al (LSF), as seen in Fig. 13. The crosslinking density of NR/AS4 increased when silica was added to the composites. It demonstrates that the hydroxyl groups on the silica’s surface prevented the rubber chains from crosslinking. It continued to be aggregated, which decreased the amount of active hydroxyl groups on the surface of the silica that could cross-link with rubber molecules^55^. The increase in Al (LSF) content significantly increases the crosslink density of the natural rubber (NR) composite structures (Fig. a & b), thereby creating a more elastic network. Furthermore, the crosslinked structure restricts natural rubber NR chain expansion during toluene immersion and reduces solvent diffusion into the intermolecular spaces in the NR matrix, ultimately decreasing the total swelling percentage.

## Conclusions

The Al(LSF) hybrid was successfully fabricated and characterized by XRF, TEM, zeta potential and EDAX techniques. Based on XRF analysis data, it was shown that lignin is the predominant component (LOI ≈ 59.98%), along with silica (22.76%) and aluminum oxide (12.16%). These results confirm the formation of a lignin-inorganic hybrid structure. TEM showed nanoparticles in the range of 39–122 nm with an average size of ~ 111 nm and relatively narrow size distribution. Hybrid particles exhibited good electrostatic stability with a low tendency to aggregate (− 28.80 mV). After incorporation into the NR matrix, the hybrid acted as a multifunctional additive which improves the curing behavior of NR and mechanical performance as well as thermo-oxidative aging resistance. Optimum hybrid loading was 1 phr, where Al(LSF) hybrid showed improved performance compared to the commercial antioxidant TMQ. Compared to silica and sodium bentonite fillers, it exhibited superior reinforcing efficiency due to enhanced crosslink density, improved tensile strength retention, higher modulus and considerably better thermo-oxidative aging resistance. Beneficial effects were achieved at low filler loading, which suggested effective filler–rubber interaction and uniform dispersion of filler in the NR matrix. Therefore, Al(LSF) hybrid displayed great promise as sustainable and eco-friendly alternative to commercial antioxidants and reinforcing fillers. Advanced composites exhibited great potential for use in different rubber products like tires, seals, gaskets, etc. and valorization of agricultural residues.

## Data Availability

All data is provided within the manuscript.
